# Physical activity and sedentary behaviour in daily life: A comparative analysis of the Global Physical Activity Questionnaire (GPAQ) and the SenseWear armband

**DOI:** 10.1371/journal.pone.0177765

**Published:** 2017-05-16

**Authors:** Michelle Laeremans, Evi Dons, Ione Avila-Palencia, Glòria Carrasco-Turigas, Juan Pablo Orjuela, Esther Anaya, Christian Brand, Tom Cole-Hunter, Audrey de Nazelle, Thomas Götschi, Sonja Kahlmeier, Mark Nieuwenhuijsen, Arnout Standaert, Patrick De Boever, Luc Int Panis

**Affiliations:** 1 Environmental Risk and Health unit (MRG), Flemish Institute for Technological Research (VITO), Mol, Belgium; 2 Transportation Research Institute (IMOB), Hasselt University, Diepenbeek, Belgium; 3 Centre for Environmental Sciences, Hasselt University, Diepenbeek, Belgium; 4 ISGlobal, Centre for Research in Environmental Epidemiology, Barcelona, Spain; 5 Universitat Pompeu Fabra, Barcelona, Spain; 6 CIBER Epidemiología y Salud Pública, Madrid, Spain; 7 Centre for Environmental Policy (CEP), Imperial College London, London, United Kingdom; 8 Transport Studies Unit, University of Oxford, Oxford, United Kingdom; 9 Department of Environmental and Radiological Health Sciences, Colorado State University, Fort Collins, Colorado, United States of America; 10 Physical Activity and Health Unit, Epidemiology, Biostatistics and Prevention Institute, University of Zurich, Zurich, Switzerland; Indiana University, UNITED STATES

## Abstract

Reduction of sedentary time and an increase in physical activity offer potential to improve public health. However, quantifying physical activity behaviour under real world conditions is a major challenge and no standard of good practice is available. Our aim was to compare the results of physical activity and sedentary behaviour obtained with a self-reported instrument (Global Physical Activity Questionnaire (GPAQ)) and a wearable sensor (SenseWear) in a repeated measures study design. Healthy adults (41 in Antwerp, 41 in Barcelona and 40 in London) wore the SenseWear armband for seven consecutive days and completed the GPAQ on the final day. This was repeated three times. We used the Wilcoxon signed rank sum test, Spearman correlation coefficients, mixed effects regression models and Bland-Altman plots to study agreement between both methods. Mixed models were used to assess the effect of personal characteristics on the absolute and relative difference between estimates obtained with the GPAQ and SenseWear. Moderate to vigorous energy expenditure and duration derived from the GPAQ were significantly lower (p<0.05) compared to the SenseWear, yet these variables showed significant correlations ranging from 0.45 to 0.64. Estimates of vigorous-intensity physical activity in particular showed high similarity (r>0.59). Results for sedentary behaviour did not differ, yet were poorly correlated (r<0.25). The differences between all variables were reproducible across repeated measurements. In addition, we observed a relationship between these differences and BMI, body fat and physical activity domain. Due to the lack of a standardized protocol, results from different studies measuring physical activity and sedentary behaviour are difficult to compare. Therefore, we suggested an easy-to-implement approach for future studies adding the GPAQ to the wearable of choice as a basis for comparisons.

## Introduction

Physical inactivity is an important modifiable risk factor for premature mortality [1]. It has been shown that sedentary behaviour (SB) and lack of physical activity (PA) independently increase the risk for metabolic syndrome [2], type 2 diabetes [3], cardiovascular disease [4] and mortality [5]. Therefore, both reducing sedentary time and increasing PA levels offer beneficial health effects, caused by different molecular and physiological mechanisms [6]. An accurate measurement of the total and intensity-specific PA level and SB in real world conditions is a crucial element in studying the association between PA, SB and health. Characterization of daily patterns of PA will enable researchers to distinguish structured exercise, SB and non-exercise PA. This is important in the context of surveillance, intervention studies and the accuracy of dose-response relationships for PA.

Quantification of PA and SB under real world conditions is an important challenge in epidemiological research [7,8]. Due to the poor quality of measurements and lack of internationally comparable data, the International Physical Activity Questionnaire (IPAQ) and the Global Physical Activity Questionnaire (GPAQ) were developed [9–11]. Both questionnaires capture information on PA duration and intensity, but the burden of filling out the longer version of the IPAQ is too high for routine surveillance. On the other hand, and contrary to the GPAQ, the short IPAQ version does not cover multiple PA domains such as work, transport and leisure time [9,10]. The GPAQ was developed by the World Health Organization (WHO) in 2002 as part of the WHO STEPwise approach to surveillance (STEPS) [9,12]. Although it performs well compared to other surveys, self-reported tools to measure PA levels are prone to sources of error such as recall bias and social desirability [9,13,14]. Sex, age, body measures, ethnicity and type of activity have been shown to influence reporting behaviour [7,13,15].

During the last decade, wearable monitors have become a key element in PA research [16]. These devices register physiological responses (e.g. heart rate) and/or mechanical bodily movements (accelerometry) [17]. Such monitors have evolved from mechanical pedometers that count steps to electronic multi-sensor devices like the SenseWear armband [17,18]. The SenseWear uses pattern recognition algorithms of various activities to provide valid estimates of PA energy expenditure [17,19,20]. The gold standard to accurately assess free-living energy expenditure is doubly labelled water (DLW), yet this method does not allow to differentiate between light-, moderate- and vigorous-intensity PA or to identify time-activity patterns [18]. In addition, measuring energy expenditure using DLW is expensive and labour-intensive. St-Onge et al. [21], Johannsen et al. [19], Mackey et al. [22] and Brazeau et al. [23] studied the difference between DLW and SenseWear results and reported a mean difference ranging from an overestimation of 25 kcal/day to an underestimation of 117 kcal/day by the SenseWear armband. They concluded that the SenseWear delivers accurate results for the measurement of total energy expenditure. Despite the advances in the field of objective monitoring, assessment of PA volume still lacks standards of good practice [16].

In this study, we used two methods to estimate PA and SB under real world conditions: the SenseWear armband, one of the most advanced wearable devices, and the GPAQ, a questionnaire developed to systematically collect data on PA levels. Both methods use a different data collection approach, yet are widely used to study the effects of activity behaviour on health. Therefore, our objectives are:

1. To determine the level of agreement between measurements of PA and SB collected with the SenseWear armband and the GPAQ.
2. To assess whether the differences between SenseWear and GPAQ estimates within a person change across repeated measures.
3. To identify personal characteristics that affect the difference between both methods.

## Materials and methods

### Study design and participants

This study was part of the FP7 PASTA project (Physical Activity through Sustainable Transport Approaches), which has been described previously [24,25]. Briefly, data on PA and travel behaviour was collected through an online survey in over 12,500 volunteers in seven European cities (Antwerp, Barcelona, London, Örebro, Rome, Vienna, Zurich). From this sample, 122 eligible and willing respondents were selected in three cities (Antwerp: 41 participants, Barcelona: 41 participants, London: 40 participants). They took part in an experiment allowing us to collect objective PA sensor data and to compare this to the subjectively reported levels of PA. Eligible participants were healthy, non-smoking, 18 to 65 year olds with a self-reported BMI below 30. Participants wore the SenseWear armband (model MF-SW, BodyMedia, USA) for seven consecutive days, only removing the sensor when there was contact with water (bathing, showering, etc.). At the end of the measurement period participants filled out the GPAQ via the online PASTA platform. Each participant repeated this procedure three times: in the mid-season, in the summer and in the winter. The repeated measurements are referred to as session 1 (mid-season), session 2 (summer) and session 3 (winter). During the participant’s last visit to the research centre, weight and percentage body fat were measured with a body composition monitor (model BF511, Omron, Japan). The study was approved by the Ethics Committee of the University hospital in Antwerp (UZA), the Comité Ético de Investigación Clíníca Parc de Salut MAR in Barcelona and the Imperial College Research Ethics Committee in London. All participants gave written informed consent prior to participation.

### Measures of PA

#### GPAQ

The GPAQ is developed and validated by the WHO to systematically monitor global PA levels [9,11]. It is included in the PASTA online questionnaire to collect information on the duration and frequency of moderate- and vigorous-intensity PA during work, transportation and leisure time (supplemental information: S1 GPAQ Questionnaire). Participants were asked only to report activities lasting 10 minutes or longer. The standard GPAQ was adjusted to ask for duration and frequency of walking, cycling and e-biking separately. Moderate/vigorous intensity activities are described as activities that require moderate/hard physical effort and cause small/large increases in breathing or heart rate. Average sitting time per day is reported as a proxy for SB. Participants filled out the GPAQ in their preferred language. Moderate-intensity activities, vigorous-intensity activities, walking, cycling and e-bike trips were assigned a value for their metabolic equivalent of task (MET) of 4, 8 [26], 4, 6.8 [27] and 5 [28,29] respectively. Cleaning of GPAQ data and calculation of moderate to vigorous METminutes per week, minutes per day and sedentary minutes per day was performed according to the WHO GPAQ analysis guidelines [26].

#### SenseWear armband

The SenseWear armband is a multi-sensor body monitor that measures heat flux, galvanic skin response, skin temperature and 3-axis accelerometry. It is worn on the tricep muscle of the left arm. Age, sex, body weight and height of the participants are provided manually to the SenseWear professional software (version 8.0). The SenseWear calculates energy expenditure and METs on a one-minute basis using proprietary algorithms based on pattern recognition [17]. For each measurement week, energy expenditure and minutes during intensity-specific activities were calculated in R version 3.3.1: moderate- to vigorous-intensity PA (MVPA), moderate-intensity PA, vigorous-intensity PA and SB. Bouts of at least 10 consecutive minutes with an intensity ≥ 3 METs were identified to match the data collection and analysis of the GPAQ [26]. If the intensity was ≥ 6 METs during at least half of the bout’s duration, it was labelled ‘vigorous-intensity’. If not, the bout was labelled ‘moderate-intensity’. Energy expenditure and minutes during moderate-intensity PA, vigorous-intensity PA and MVPA were calculated as the sum of respective METs and minutes during the identified bouts. Minutes of SB per day are calculated as the sum of minutes with METs ≤ 1.5 minus time spent sleeping [26,30–32].

### Statistical analysis

Medians and interquartile ranges (IQR) were reported because the calculated variables were not normally distributed. PA measurements collected with GPAQ and SenseWear during each session were compared using the Wilcoxon signed rank sum test and Spearman correlation. To assess the overall level of agreement, Spearman correlation coefficients adjusted for repeated observations were calculated according to Bland & Altman [33]. These overall correlation coefficients could not be tested for significance. For the interpretation of the Spearman correlation coefficients, we used the scale defined by Landis & Koch [34].

To study individual differences, Bland-Altman plots were constructed using 95% limits of agreement adjusted for repeated measures according to Bland & Altman [35]. Absolute differences showed an increase in variability when the magnitude of the PA variables increased. Therefore, the percentage difference Bland-Altman plots were used. However, in the GPAQ, 0 minutes of moderate- and vigorous-intensity PA were reported 43 and 50 times, respectively. Furthermore, 0 minutes of vigorous-intensity PA were measured by the SenseWear 25 times. These are influential observations and cause deviations from the normal distribution of the differences. Consequently, the mean difference and 95% limits of agreement were calculated both with and without these observations. When the limits of agreement exceeded 200%, the largest possible percentage difference, the limit was drawn at a percentage difference of 200. In addition, for vigorous-intensity PA, 0 minutes were both reported in the GPAQ and measured by the SenseWear 40 times. This results in an undefined percentage difference. In order to include these observations in the calculation of the mean difference and 95% limits of agreement, we used 0.001 instead.

Mixed effect regression models were used to study: (1) whether the difference between GPAQ and SenseWear measurements changed per session (Δ(t) model; Table 1), and; (2) the effect of participant characteristics on the difference between both methods (attribute models; Table 1). For the latter objective, we used separate models to study the effect of sex, age, BMI, percentage body fat and PA domain as a descriptive analysis. We assumed that BMI and percentage body fat were stable during the observation period. Consequently, inclusion of all measurement weeks increased our statistical power. For each measurement week, the dominant PA domain (work, transport or leisure-time) was determined using the GPAQ data. This was based on the assumption that the participants’ recall bias depended on the PA domain in which they were most active. All mixed models included random participant effects nested in city effects.

**Table 1:** Mixed effect regression models used to analyse (1) the change in difference between GPAQ and SenseWear measurements; (2) the effect of personal attributes on the difference between both methods.

**Δ(t) model**	Δ_*ijk*_ = *β*_0_ + *a*_*i*_ + *b*_*j* (*i*)_ + *β*_1_*session* 2_*ijk*_ + *β*_2_*session* 3_*ijk*_ + *ε*_*ijk*_
**Attribute models**	Δ_*ijk*_ = *β*_0_ + *a*_*i*_ + *b*_*j* (*i*)_ + *β*_1_*attribute*_*ijk*_ + *ε*_*ijk*_
**Assumptions**	$a_{i}∼N(0,\sigma_{cities}^{2})$; $b_{ij}∼N(0,\sigma_{participants}^{2})$; $\varepsilon_{ijk}∼N(0,\sigma_{residuals}^{2})$

Where Δ_*ijk*_ is the difference between the estimates of both methods for the k^th^ measurement of individual j in city i.

## Results

A total of 122 healthy adults took part in the study between February 2015 and March 2016 (45% males, 89% with higher education degrees, 94% Caucasian, age: 35 ± 10 years, BMI: 24 ± 3 kg/m^2^; S1 Table). 119 participants completed three 7-day measurement periods (1 participant participated during 2 periods; 2 participants completed only 1 period). Out of 361 GPAQ and SenseWear estimates per variable respectively 11 (reported time was invalid) and 3 (failed read-out and incorrect wearing) were missing. Consequently, 16 participants had less than 3 pairs of data (13 had 2 pairs; 3 had 1 pair). The SenseWear armband was worn 96 ± 4% of the time (average and SD of the average wearing time per individual).

Table 2 shows the results from both measurement methods aggregated over three sessions per individual. We recruited an active sample: only 13% (GPAQ) and 7% (SenseWear) of METminutes/week were below 600 i.e. the WHO recommendation on PA [30]. This corresponds to 5% (GPAQ) and 2% (SenseWear) of our participants having a PA level below the recommendation during all sessions.

**Table 2:** Median and IQR of the PA measures aggregated over three sessions per participant for both measurement methods (GPAQ and SenseWear). Number of participants included in the analysis is 122.

	MVPA EE (METmin/week)	Moderate EE (METmin/week)	Vigorous EE (METmin/week)	MVPA (min/day)	Moderate PA (min/day)	Vigorous PA (min/day)	SB (min/day)
**GPAQ (median (IQR))**	2029 (1112–3237)	720 (310–1268)	1057 (321–2169)	53 (33–78)	26 (11–45)	22 (6–40)	535 (420–635)
**SenseWear (median (IQR))**	2569 (1688–4280)	1575 (1133–2256)	807 (207–2154)	71 (49–111)	47 (34–67)	17 (4–44)	550 (481–653)
**% Difference** ^a^ **(median (IQR))**	%						
	39 (0–75)	77 (30–144)	7 (-56-69)	34 (0–79)	62 (9–136)	7 (-53-71)	8 (-12-30)

GPAQ = global physical activity questionnaire, MVPA = moderate to vigorous physical activity, EE = energy expenditure, SB = sedentary behaviour

^a^ The percentage difference was calculated by subtracting GPAQ from SenseWear results divided by their average and reported as the median and IQR of the average difference per participant.

**Fig 1:**
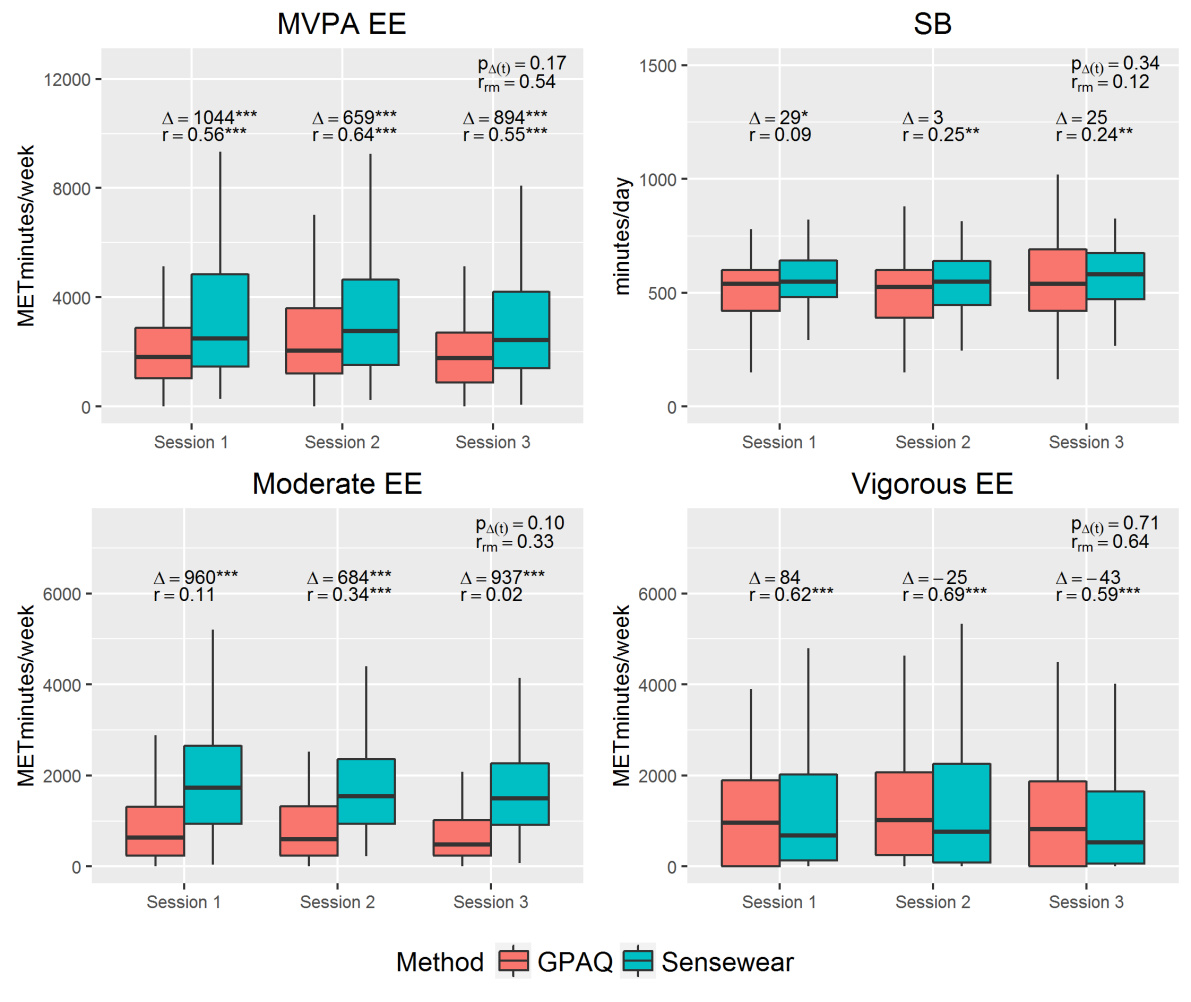
Boxplots of MVPA energy expenditure (EE), SB, moderate EE and vigorous EE per method and session. For each session, the difference Δ and Spearman correlation coefficient r is specified. Δ was calculated as the mean difference between both methods and was tested for significance using the Wilcoxon signed rank sum test. r_rm_ is the overall Spearman correlation adjusted for repeated measures (rm) [33]; p_Δ(t)_ = the p-value of the effect of session in the Δ(t) model which indicates if the difference between GPAQ and SenseWear measurements changes per repeated measurement. Statistical significance is expressed as *p<0.05, **p<0.01, and *** p<0.001.

Estimates for moderate to vigorous and moderate energy expenditure obtained by the GPAQ were significantly lower compared to the SenseWear (Fig 1). However, no statistically significant differences between both methods were observed for vigorous energy expenditure alone. Measures of overall MVPA and vigorous energy expenditure from the GPAQ and SenseWear were significantly correlated. Moderate energy expenditure only showed a significant correlation on session 2. Moderate, MVPA and vigorous energy expenditure had an overall Spearman correlation (r_rm_) of fair, moderate and high strength, respectively. The differences between both methods were reproducible as they did not change over time (i.e. no significant overall effect of session in the Δ(t) model; p_Δ(t)_ > 0.05). The same trends were observed for minutes per day of MVPA, moderate- and vigorous-intensity PA (supplemental information: S1 Fig).

Sedentary minutes measured by the GPAQ and SenseWear were significantly different on session 1 only (Fig 1). SB was poorly correlated between both methods. Hence, for each amount of sedentary minutes measured by the SenseWear, we observed a large range of estimates reported in the GPAQ (supplemental information: S2 Fig). Similarly to the results for PA variables, the difference in SB between both methods did not change significantly across sessions.

**Fig 2:**
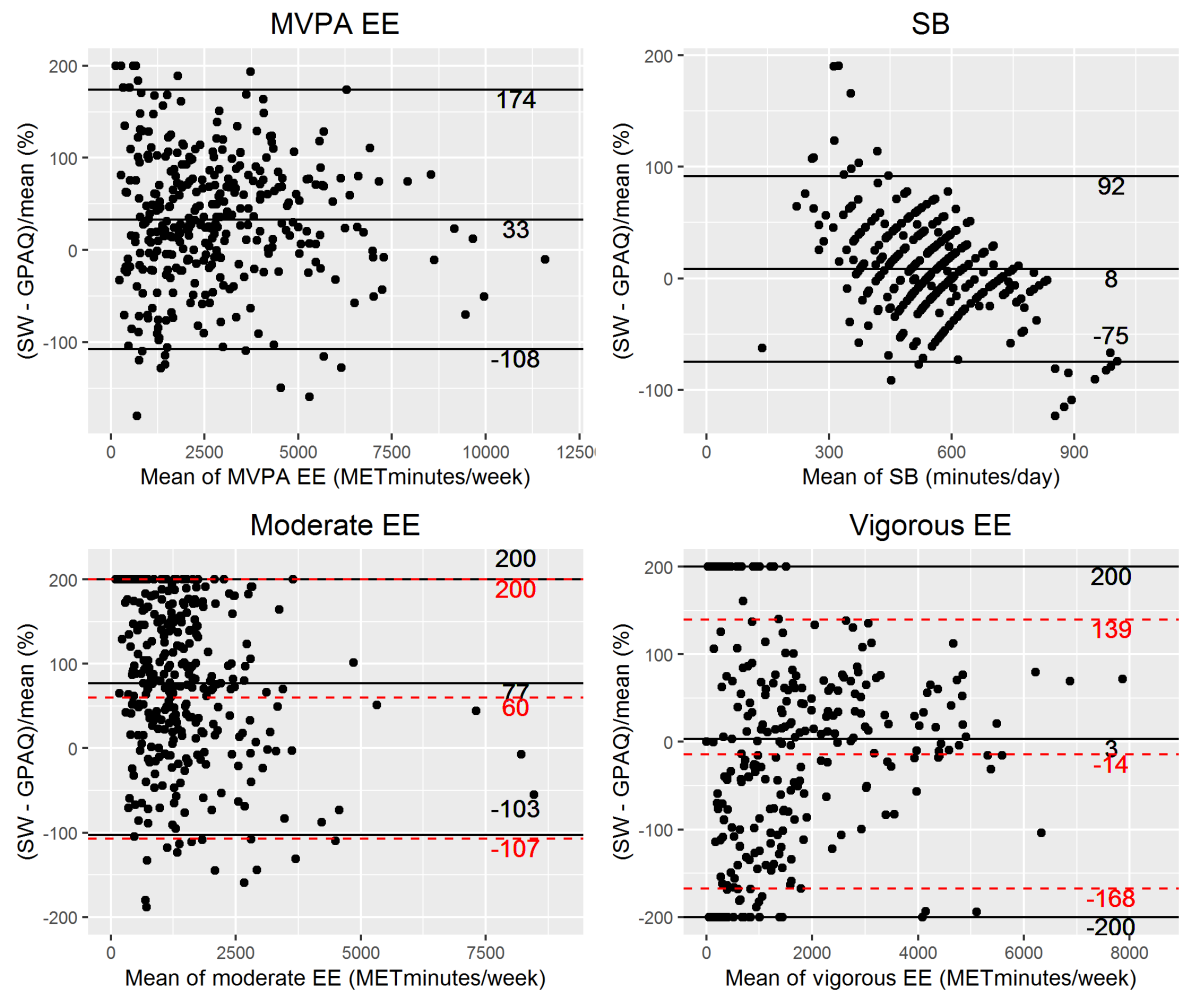
Bland-Altman plots comparing MVPA, moderate and vigorous energy expenditure (METminutes/week) and SB (minutes/day) measured by the SenseWear armband (SW) and the GPAQ. All percentage differences on the Y-axis are calculated by subtracting GPAQ from SenseWear results divided by their average. Moderate- and vigorous-intensity activities included influential observation. The red, dashed lines represent the mean difference and 95% limits of agreement excluding these observations. EE = energy expenditure.

Bland-Altman plots using the percentage difference further illustrate that lower levels of moderate to vigorous and moderate energy expenditure were obtained with the GPAQ, while the mean difference for vigorous energy expenditure was close to 0 (Fig 2). On the other hand, the 95% limits of agreement were wide. For moderate and vigorous energy expenditure, 3 out of 4 limits reached the maximum percentage difference of 200. This was due to the inclusion of influential observations where either the GPAQ or SenseWear estimate was 0. Excluding these observations still resulted in limits where the magnitude of the differences exceeded the size of the mean difference. Analysis of MVPA, moderate- and vigorous-intensity PA minutes per day showed similar results (supplemental information: S3 Fig).

The Bland-Altman plot for SB indicates a mean difference close to 0 and narrower 95% limits of agreement. However, a relationship between the differences and the mean was observed. When the amount of SB increased, the amount of sedentary time reported in the GPAQ exceeded the estimate of the SenseWear.

The discrepancy between SenseWear and GPAQ estimates was not affected by sex or age (Table 3). Per unit increase in BMI and percentage body fat, the absolute difference decreased significantly for MVPA and vigorous-intensity PA. For vigorous-intensity PA, similar, non-significant trends were observed for the percentage differences. Furthermore, participants mainly active during work compared to those mainly active during leisure time had significantly lower differences between SenseWear and GPAQ estimates for MVPA and moderate-intensity PA. For vigorous-intensity activities and MVPA, the percentage differences increased significantly for participants mainly active during transport compared to leisure time. Regarding the differences between both methods in SB, we observed the opposite trends for BMI and body fat compared to the estimates of activity. For PA domain, both the absolute and percentage difference increased for SB in participants mainly active during work.

**Table 3:** β_1_-coefficients of the attribute models to indicate the effect of sex, age, BMI, body fat and PA domain on the absolute and percentage difference (Δ) between measured (SenseWear) and reported (GPAQ) METminutes/week, minutes PA/day and sedentary minutes/day. The difference between both methods is used as the dependent variable. Absolute differences were calculated as SenseWear minus GPAQ results. Percentage differences are calculated by dividing the absolute difference by the average of the measurements from both methods. Separate models were fitted for each characteristic. Each model included random participant effects clustered per city.

*β*_1_ ± SE		Sex ^a^	Age	BMI	Body fat	PA domain ^b^	
			years	kg/m^2^	%	Transport	Work
**Δ MVPA EE**	**METminutes/week**	431.7±344.7	-6.12±17.59	-151.57±55.49**	-57.04±20.84**	153.9±277.5	-1370.4±521.8**
	**%**	6.12±10.7	-0.47±0.54	-2.64±1.73	-0.43±0.66	21.24±8.47*	-38.14±15.91*
**Δ Moderate EE**	**METminutes/week**	27.78±233.52	-9.75±11.8	-51.50±38.32	-13.57±14.47	-53.13±200.13	-1136.84±368.99**
	**%**	-5.9±13.96	-0.99±0.70	-2.35±2.29	-0.32±0.87	1.68±10.87	-78.25±20.28***
**Δ Vigorous EE**	**METminutes/week**	400.84±223.6	3.37±11.5	-99.25±36.31**	-43.62±13.47**	320.83±218.18	20.41±396.06
	**%**	30.32±16.41	-1.01±0.84	-5.15±2.68	-1.91±1.01	40.76±15.68**	33.27±28.58
**Δ MVPA**	**minutes/day**	4.73±8.91	-0.24±0.45	-2.96±1.45*	-0.93±0.55	1.42±7.36	-48.36±13.69***
	**%**	0.31±10.92	-0.51±0.55	-1.96±1.78	-0.13±0.67	14.45±8.38	-52.29±15.77**
**Δ Moderate PA**	**minutes/day**	-2.36±7.45	-0.29±0.38	-0.97±1.23	-0.10±0.46	-1.54±6.45	-47.82±11.79***
	**%**	-7.15±14.43	-1.01±0.73	-1.6±2.38	-0.09±0.9	2.04±11.20	-83.03±20.88***
**Δ Vigorous PA**	**minutes/day**	6.99±4.20	0.04±0.22	-1.96±0.68**	-0.83±0.25**	4.71±4.21	2.1±7.58
	**%**	28.32±16.34	-1.05±0.83	-5.25±2.66	-1.84±1	38.44±15.66*	31.53±28.52
**Δ SB**	**minutes/day**	-50.9±37.35	1.05±1.91	12.46±6.03*	5.03±2.26*	-15.19± 25.64	123.45±49.18*
	**%**	-9.32±6.75	0.11±0.34	1.77±1.1	0.82±0.41 *	-7.06±4.47	32.55±8.57***

^a^ women are the reference category

^b^ leisure time PA is the reference category

Statistical significance is expressed as

*p<0.05

**p<0.01, and

*** p<0.001

## Discussion

This is a comparative analysis of PA and sedentary levels measured by the GPAQ and the SenseWear armband. Compared to previous studies, we collected a large dataset using a repeated measures design on 122 participants in three different European cities [36–39] The estimates obtained with the GPAQ were significantly lower for MVPA and moderate-intensity PA; no difference was observed for vigorous-intensity activities and SB. Significant positive correlations were found for MVPA and vigorous-intensity PA and the overall Spearman correlation was of respective moderate and high strength. Non-significant poor to fair correlations were observed for SB and moderate-intensity PA.

Cleland et al. [36] compared the GPAQ to the Actigraph and did not find a significant difference for MVPA time, while SB did differ between both methods. Though, similar to our results, moderate [36] and fair [40] correlations were reported for MVPA time, and poor agreement for SB [36]. The low correlation for SB could be explained by the use of a single item to measure sedentary time in the GPAQ. Hence, including multiple, domain-specific items to assess SB is needed as recent papers stress the importance of SB for public health [41].

Other studies compared the SenseWear armband to an interviewer-administered 24h PA recall (PAR) [14] and the Flemish Physical Activity Computerized Questionnaire (FPACQ) [7]. The time between the activity and recall is only 24 hours when using PAR, compared to seven days for the GPAQ, which results in lower recall bias [13,42]. Similar to our results, both studies reported moderate to high correlations for total energy expenditure and MVPA time.

Our participants reported more vigorous-intensity PA compared to moderate-intensity PA. The tendency towards overestimating vigorous activity and underreporting moderate activity has already been shown [7,37,38,43–45]. Possibly, participants perceive or recall the exertion different than the objective physical output. This makes it difficult to distinguish between moderate and vigorous intensity when reporting PA levels [10,43]. However, when reporting PA, respondents think about vigorous or organized activities such as sports, while they forget about moderate, routine activities (e.g. household chores or active transportation) or incidental daily movements [7]. This might explain the better agreement between the GPAQ and the Sensewear for vigorous energy expenditure and time. Besides, it illustrates the importance of reporting domain specific activities.

Accordingly, we found a significant effect of PA domain on the difference between the SenseWear and GPAQ estimates. When looking at percentage differences, the amount of physical activity engaged in is filtered out. Consequently, participants mainly active during transport compared to leisure-time had a similar activity level, yet the difference between both methods increased for MVPA energy expenditure and vigorous-intensity activities. Hence, besides underreporting moderate, routine activities, we hypothesise that vigorous, routine activities such as cycling are underreported as well. In addition, the difference was significantly smaller for MVPA and moderate-intensity activities in participants mainly active during work compared to leisure time. Other studies comparing PA questionnaires to more objective measurement techniques also found higher correspondence for reported PA during work compared to leisure time or transport [46,47]. In our study with participants with a BMI below 30, we observed an effect of BMI and body fat on the difference between estimates of the SenseWear and GPAQ. We found that the absolute differences for MVPA energy expenditure and time decreased per unit increase in BMI and percentage body fat, similar to Welk et al. [14]. In addition, we observed the opposite effect for SB. However, when the percentage differences were tested, the effect did not remain for activity measures due to the association between PA level as such and BMI or percentage body fat. When studying the effects of BMI and body fat on moderate- and vigorous- intensity PA separately, we observed a (non-significant) decrease in the difference for vigorous-intensity PA for both the absolute and percentage differences. Therefore, we hypothesise that heavier participants report more organized PA and less SB due to social desirability. On the other hand, METs, as measured by the SenseWear, do not take the perceived rate of exertion into account [8]. Hence, an activity experienced as vigorous by an overweight person could be classified as moderate by the SenseWear. Finally, contrary to previous work, we did not find an effect of sex and age on MVPA energy expenditure and time [7,14].

Bland-Altman plots showed a wide range of individual differences between GPAQ and SenseWear measurements for all variables, which agrees with previous work [7,10,36]. This indicates that the GPAQ was unable to assess differences on an individual level, yet it was not developed for this purpose [11].

A major strength of our study is the use of repeated measures. We observed a reproducible difference between the estimates of both methods. To our knowledge only Cleland et al. [36] assessed the validity of GPAQ in measuring changes in PA level and SB. The authors suggested that the GPAQ provides reliable results to assess change in MVPA time while it didn’t for SB. The results from our study and those of Cleland et al. [36] show the GPAQ to be an easy-to-use and inexpensive instrument to monitor levels of change in PA on a population level.

In contrast to other studies, we obtained a significantly higher level of overall and moderate-intensity PA with the SenseWear compared to the self-reported instrument [7,38]. We suggest the following explanations for this observation:

1. Because our participants were highly active, we hypothesise that they were unable to recall all moderate activities that were part of their daily habits. Only 2 to 5% did not meet the PA recommendation of 600 METminutes/week, while the inactivity level of the global population is 31% [11]. Similarly to our study, Welk et al. [14] used the SenseWear armband (software version 8.0) and recruited a highly active sample (MVPA time: 131 minutes/day). They also obtained higher values of total energy expenditure with the SenseWear compared to the used self-reported instrument. However, on average, total energy expenditure was 10% higher when using the Sensewear. This is lower than our observation of 33%.
2. Another possible explanation is based on the variation in performance of different wearable sensors. Both Actigraph and SenseWear are widely used by researchers to measure PA and SB [39]. Both methods overestimate time in MVPA. This might explain why Cleland et al. [36] reported a non-significant overestimation of MVPA time per day with the Actigraph (56 minutes/day) compared to the GPAQ (30 minutes/day). In addition, Actigraph has been shown to underestimate energy expenditure during MVPA while SenseWear overestimates/ underestimates energy expenditure during moderate-/ vigorous-intensity PA, respectively [20,48]. Moreover, contrary to Actigraph and other hip-worn accelerometers, the SenseWear armband uses activity pattern recognition to estimate energy expenditure. This results in a more accurate measure of upper body movements or cycling, while hip-worn accelerometers don’t register these movements [7,17,20,36,37]. This could lead to higher results obtained with the SenseWear compared to the Actigraph and other hip-worn accelerometers.
3. Our last hypothesis is based on the low wearing time of most accelerometers [7,37]. In our study, the SenseWear was worn during waking and sleeping hours and there were no battery issues. This resulted in a wearing time of 96%. Consequently, we were able to track movement during periods of time when other activity monitors are not worn (before and during the night, early mornings etc.).

A review by Prince et al. [43] found that there is no consensus about the trends in level of agreement between self-reported instruments and wearables. Results of comparisons between self-reported and measured PA differ depending on the characteristics of the wearable, the questionnaire and the sample [43,49]. When comparing wearables and self-reported tools, the intensity assigned to reported activities never equals the intensity measured by the wearable. This is an important limitation. According to the WHO GPAQ analysis guide, we assigned 4 and 8 METs to moderate- and vigorous- PA respectively. The SenseWear measured on average 4.62/ 6.72 METs for moderate/ vigorous PA bouts of at least 10 minutes. This impacts the comparability of MVPA, moderate and vigorous energy expenditure, yet does not affect the comparability of durations. Furthermore, a lot of wearable sensors use proprietary algorithms to calculate energy expenditure [16,50]; this limits the possibilities to compare results of different studies. To illustrate, the SenseWear armband uses proprietary algorithms based on activity pattern recognition to calculate energy expenditure and METs. On the other hand, the Actigraph reports proprietary counts which cause confusion for the conversion into estimates of PA and the interpretation of results [19]. Clearly, in order to enable a systematic evaluation of PA levels and SB, it is advised to limit the use of such proprietary algorithms.

The quest for a standardized protocol to measure PA and SB is still ongoing and the use of one standard wearable in all studies is challenging [43]. To enable both characterisation of daily movement patterns and comparison of PA data across studies, we suggest to use both the GPAQ, a standardized and generally accepted questionnaire, and a wearable meeting the specific needs of the study. A questionnaire is practical, budget-friendly and has a low participant burden. Systematic use of the GPAQ, a WHO validated questionnaire, in addition to a wearable offers a basis for the comparison of different studies measuring PA and SB. Such an approach could help assess and disentangle the different factors causing variation between PA estimates of different methods e.g. recall bias due to sample characteristics or study design and performance of specific wearables.

## Conclusion

PA estimates obtained with the GPAQ were significantly lower compared to those obtained using the SenseWear armband. MVPA energy expenditure and time were moderately correlated between both methods and SB was poorly correlated. In contrast to moderate-intensity PA, estimates of both methods highly agreed for vigorous-intensity PA.

We observed reproducible differences between the estimates of both methods. Thus, the GPAQ is an acceptable tool to measure levels of change in the population’s activity behaviour. We also found associations between the difference in results and BMI, body fat and PA domain. The strength of these associations depended on the intensity of the activity which emphasizes the importance of analyzing sedentary, moderate- and vigorous-intensity PA separately.

## Supporting information

S1 Dataset Dataset including the relevant data without demographic information. –Demographic information was not added due to privacy concerns.
(XLSX)

S1 Table Characteristics of volunteers enrolled in 1) the PASTA online survey in all cities 2) the PASTA online survey in Antwerp (ANT), Barcelona (BCN) and London (LDN) only 3) the study using wearables (all participants and each city separately). Physical activity variables of the online survey sample are derived from the GPAQ asking about general behaviour. PA variables of the subset enrolled in the study using wearables are derived from the GPAQ asking about activities during their measurement week.
(PDF)

S1 GPAQ Questionnaire The Global Physical Activity Questionnaire (GPAQ). The GPAQ was adjusted to capture information on walking, cycling and e-biking trips separately.
(PDF)

S1 Fig Boxplots of MVPA time, moderate time and vigorous time per measurement method and session. Δ = the mean difference between both methods per session (tested for significance using the Wilcoxon signed rank sum test); r = the Spearman correlation coefficient per session; r_rm_ = the overall Spearman correlation adjusted for repeated measures (rm); p_Δ(t)_ = the p-value of the effect of session in the Δ(t) model which indicates if the difference between GPAQ and SenseWear measurements changes over time or sessions. Statistical significance is expressed as *p<0.05, **p<0.01, and *** p<0.001.
(PDF)

S2 Fig Sedentary minutes measured by the GPAQ in function of SB measured by the SenseWear. The Spearman correlation coefficients for session 1, session 2 and session 3 are respectively 0.09, 0.25 and 0.24 (overall r_rm_ = 0.12). SW = SenseWear.
(PDF)

S3 Fig Bland-Altman plots comparing MVPA, moderate and vigorous time (minutes/week) measured by the SenseWear armband (SW) and the GPAQ. All percentage differences on the Y-axis are calculated by subtracting GPAQ from SenseWear results divided by their average. Moderate and vigorous intensity activities included influential observation. The red, dashed lines represent the mean difference and 95% limits of agreement excluding these observations.
(PDF)
