# Supplementary material for: Physical activity and sedentary behaviour in daily life: A comparative analysis of the Global Physical Activity Questionnaire (GPAQ) and the SenseWear armband
Source: PLoS One. 2017 May 16;12(5):e0177765. doi: 10.1371/journal.pone.0177765 (PMC5433749; doi:10.1371/journal.pone.0177765)
Supplement: S1 Table — Physical activity variables of the online survey sample are derived from the GPAQ asking about general behaviour. PA variables of the subset enrolled in the study using wearables are derived from the GPAQ asking about activities during their measurement week. (PDF) [file pone.0177765.s002.pdf]

S1 Table Characteristics of volunteers enrolled in 1) the PASTA online survey in all cities 2) the PASTA online survey in Antwerp (ANT), Barcelona (BCN) and London (LDN) only 3) the study using wearables (all participants and each city separately). Physical activity variables of the online survey sample are derived from the GPAQ asking about general behaviour. PA variables of the subset enrolled in the study using wearables are derived from the GPAQ asking about activities during their measurement week.

		PASTA online survey	PASTA online survey ANT,BCN,LDN	Subset used for analysis ANT, BCN, LDN	Subset used for analysis ANT	Subset used for analysis BCN	Subset used for analysis LDN
**n**		10693*	4618*	122	41	41	40
**Males**	%	46	43	45	56	39	40
**Higher education**	%	78	86	89	90	90	88
**Age (mean ± sd)**	years	40 ± 13	39 ± 13	35 ± 10	37 ± 11	34 ± 9	35 ± 10
**Reported BMI (mean ± sd)**	kg/m²	24 ± 4	24 ± 4	23 ± 3	23 ± 3	23 ± 3	22 ± 3
**MVPA EE (median (IQR))**	METminutes /week	2820 (1560-4857)	2720 (1468-4717)	2029 (1112-3237)	1972 (1251-3271)	1600 (1000-2232)	2523 (1408-3334)
**Moderate EE (median (IQR))**	METminutes /week	960 (360-2280)	960 (240-2160)	720 (310-1268)	360 (120-850)	760 (413-1213)	870 (560-1580)
**Vigorous EE (median (IQR))**	METminutes /week	1440 (320-2880)	1410 (272-2796)	1057 (321-2169)	1632 (752-2285)	408 (107-1387)	1184 (147-2330)
**MVPA (median (IQR))**	minutes/day	73 (40-129)	69 39-122)	53 (33-78)	46 (31-78)	43 (30-66)	64 (40-90)
**Moderate MVPA (median (IQR))**	minutes/day	34 (13-81)	34 (9-77)	26 (11-45)	13 (4-30)	27 (15-43)	31 (20-56)
**Vigorous MVPA (median (IQR))**	minutes/day	26 (6-54)	26 (6-51)	22 (6-40)	30 (15-43)	9 (2-28)	23 (3-45)
**SB (median (IQR))**	minutes/day	480 (330-600)	480 (330-600)	535 (420-635)	460 (390-580)	590 (480-720)	520 (415-600)

MVPA = moderate to vigorous physical activity, EE = energy expenditure, SB = sedentary behaviour
* cleaned data
