# Supplementary material for: Physical activity and sedentary behaviour in daily life: A comparative analysis of the Global Physical Activity Questionnaire (GPAQ) and the SenseWear armband
Source: PLoS One. 2017 May 16;12(5):e0177765. doi: 10.1371/journal.pone.0177765 (PMC5433749; doi:10.1371/journal.pone.0177765)
Supplement: S1 GPAQ Questionnaire — The GPAQ was adjusted to capture information on walking, cycling and e-biking trips separately. (PDF) [file pone.0177765.s003.pdf]

Finish line Q

The finish line

4%

Hi again!

We will repeat the same procedure as during the kick-off session. Please make sure that you are wearing the heart rate monitor (Zephyr BioHarness).

Again, we will start by asking you some questions about the course of the measurement week.

Next

The finish line

9%

Activity at work

Think of work as the things that you have to do such as paid or unpaid work, study/training, and household chores or gardening.

Vigorous-intensity activities are activities that require hard physical effort and cause large increases in breathing or heart rate.

Moderate-intensity activities are activities that require moderate physical effort and cause small increases in breathing or heart rate.

Does your work involve vigorous-intensity activities for at least 10 minutes continuously? more info

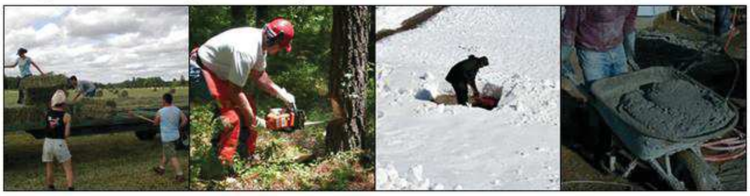

(•) Yes
( ) No

In the last 7 days, on how many days did you do vigorous-intensity activities as part of your work?

2

Typically, how much time do you spend doing vigorous-intensity activities at work on such a day?

1:00

Please enter the duration as hours:minutes, separated by ":" (e.g., 2:30).

Does your work involve moderate-intensity activity for at least 10 minutes continuously? more info

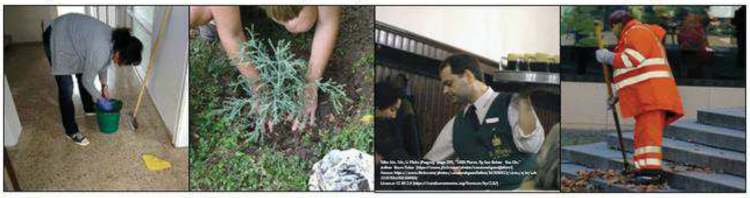

( ) Yes
(•) No

Previous

Next

The finish line

13%

Travel to and from places

The following questions exclude the physical activities at work that you have already mentioned.

Now think about the usual way in which you travel to and from places. Do *not* include walking or cycling for leisure or sports.

In the last 7 days, on how many days did you use each of the following methods of travel to get to and from places? more info

	Did not use it	on 1-3 days per week	on 4-5 days per week	on 6-7 days per week
Walk	○	○	●	○
Bicycle	●	○	○	○
Electric bicycle	●	○	○	○
Motorcycle or moped	●	○	○	○
Public transport	○	●	○	○
Car or van	○	●	○	○

In the last 7 days, did you walk or use a bicycle for at least 10 minutes continuously to get to and from places?

- [x] Walk
- [ ] Bicycle
- [ ] Electric bicycle
- [ ] No

In the last 7 days, on how many days did you walk for at least 10 minutes continuously to get to and from places?

5

Typically, how much time do you spend walking on such a day?

0:40

Please enter the duration as hours:minutes, separated by ":" (e.g., 2:30).

Previous   Next

The finish line

18%

Recreational activities

For the next questions exclude the work and transport activities that you have already mentioned. Now think about sports, fitness and recreational activities (leisure), including going for a walk or on a cycle tour.

Vigorous-intensity activities are activities that require hard physical effort and cause large increases in breathing or heart rate.

Moderate-intensity activities are activities that require moderate physical effort and cause small increases in breathing or heart rate.

Do you do any vigorous-intensity sports, fitness or recreational (leisure) activities for at least 10 minutes continuously? more info

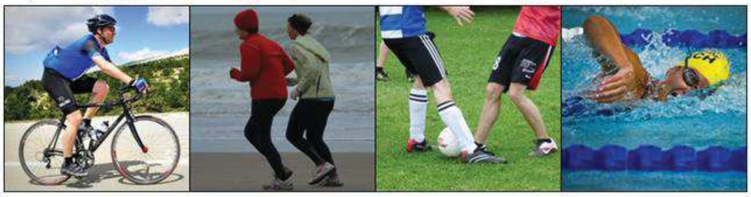

- ○ Yes
- ◉ No

Do you do any moderate-intensity sports, fitness or recreational (leisure) activities for at least 10 minutes continuously? more info

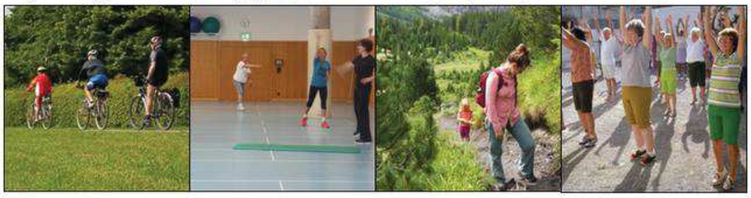

- ◉ Yes
- ○ No

In the last 7 days, on how many days did you do moderate-intensity sports, fitness or recreational (leisure) activities?

1

Typically, how much time do you spend doing moderate-intensity sports, fitness or recreational activities on such a day?

1:30

Please enter the duration as hours:minutes, separated by ":" (e.g., 2:30).

Previous | Next

The finish line

22%

Sedentary behaviour

The following question is about sitting or reclining at work, at home, getting to and from places, or with friends. Time spent sleeping should not be included.

For example: time spent sitting at a desk; eating; travelling in car, bus or train; reading; watching television; or using the computer.

In the last 7 days, how much time did you spend sitting or reclining on a typical day?

8:30

Please enter the duration as hours:minutes, separated by ":" (e.g., 2:30).

Previous | Next
