# Supplementary material for: Physical activity and sedentary behaviour in daily life: A comparative analysis of the Global Physical Activity Questionnaire (GPAQ) and the SenseWear armband
Source: PLoS One. 2017 May 16;12(5):e0177765. doi: 10.1371/journal.pone.0177765 (PMC5433749; doi:10.1371/journal.pone.0177765)
Supplement: S1 Fig — Δ = the mean difference between both methods per session (tested for significance using the Wilcoxon signed rank sum test); r = the Spearman correlation coefficient per session; rrm = the overall Spearman correlation adjusted for repeated measures (rm); pΔ(t) = the p-value of the effect of session in the Δ(t) model which indicates if the difference between GPAQ and SenseWear measurements changes over time or sessions. Statistical significance is expressed as *p<0.05, **p<0.01, and *** p<0.001. (PDF) [file pone.0177765.s004.pdf]

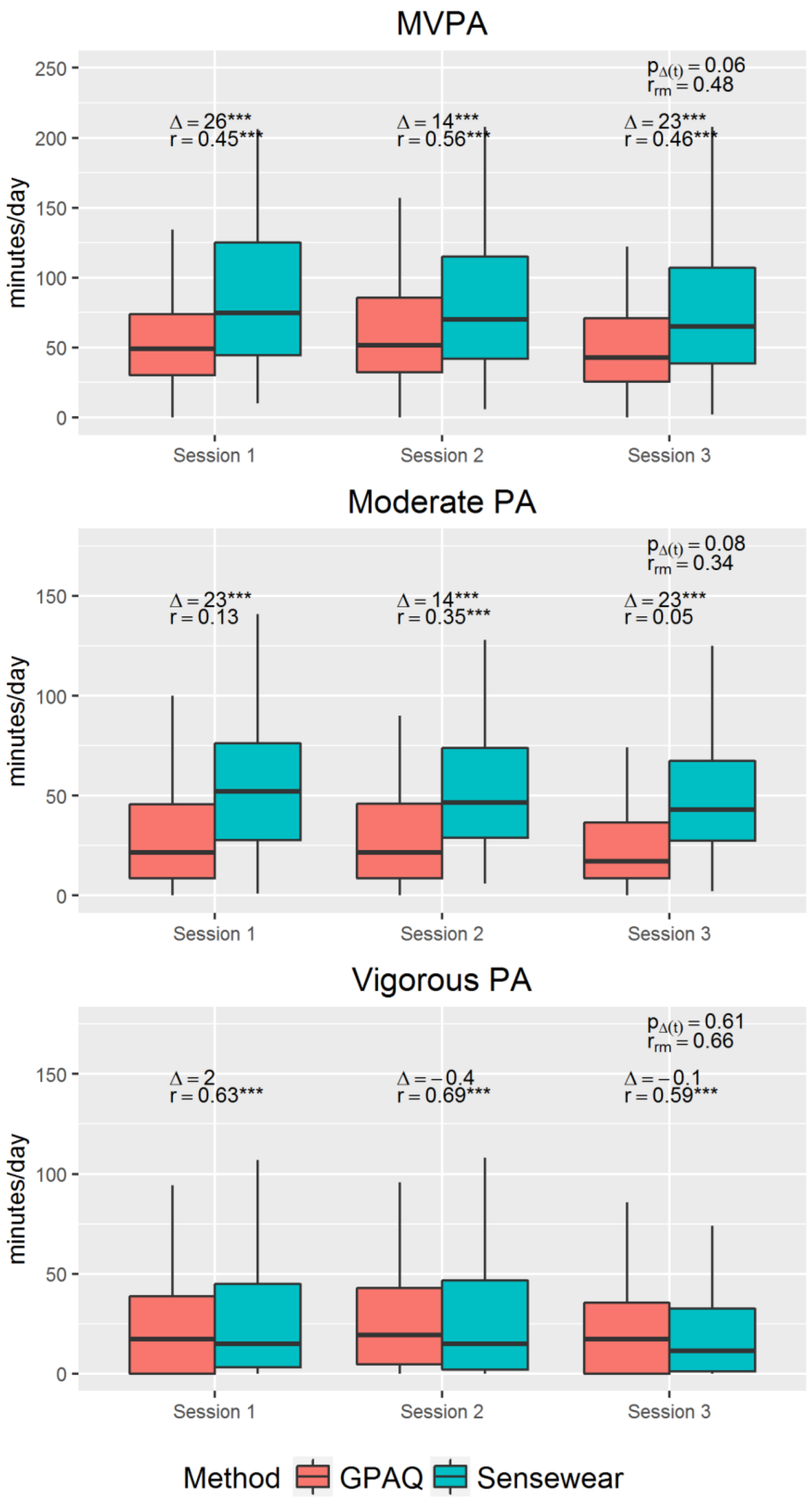

S1 Fig Boxplots of MVPA time, moderate time and vigorous time per measurement method and session. Δ= the mean difference between both methods per session (tested for significance using the Wilcoxon signed rank sum test); r = the Spearman correlation coefficient per session; rrm = the overall Spearman correlation adjusted for repeated measures (rm); pΔ(t) = the p-value of the effect of session in the Δ(t) model which indicates if the difference between GPAQ and SenseWear measurements changes over time or sessions. Statistical significance is expressed as *p<0.05, **p<0.01, and *** p<0.001
