# Supplementary material for: Physical activity and sedentary behaviour in daily life: A comparative analysis of the Global Physical Activity Questionnaire (GPAQ) and the SenseWear armband
Source: PLoS One. 2017 May 16;12(5):e0177765. doi: 10.1371/journal.pone.0177765 (PMC5433749; doi:10.1371/journal.pone.0177765)
Supplement: S2 Fig — The Spearman correlation coefficients for session 1, session 2 and session 3 are respectively 0.09, 0.25 and 0.24 (overall rrm = 0.12). SW = SenseWear. (PDF) [file pone.0177765.s005.pdf]

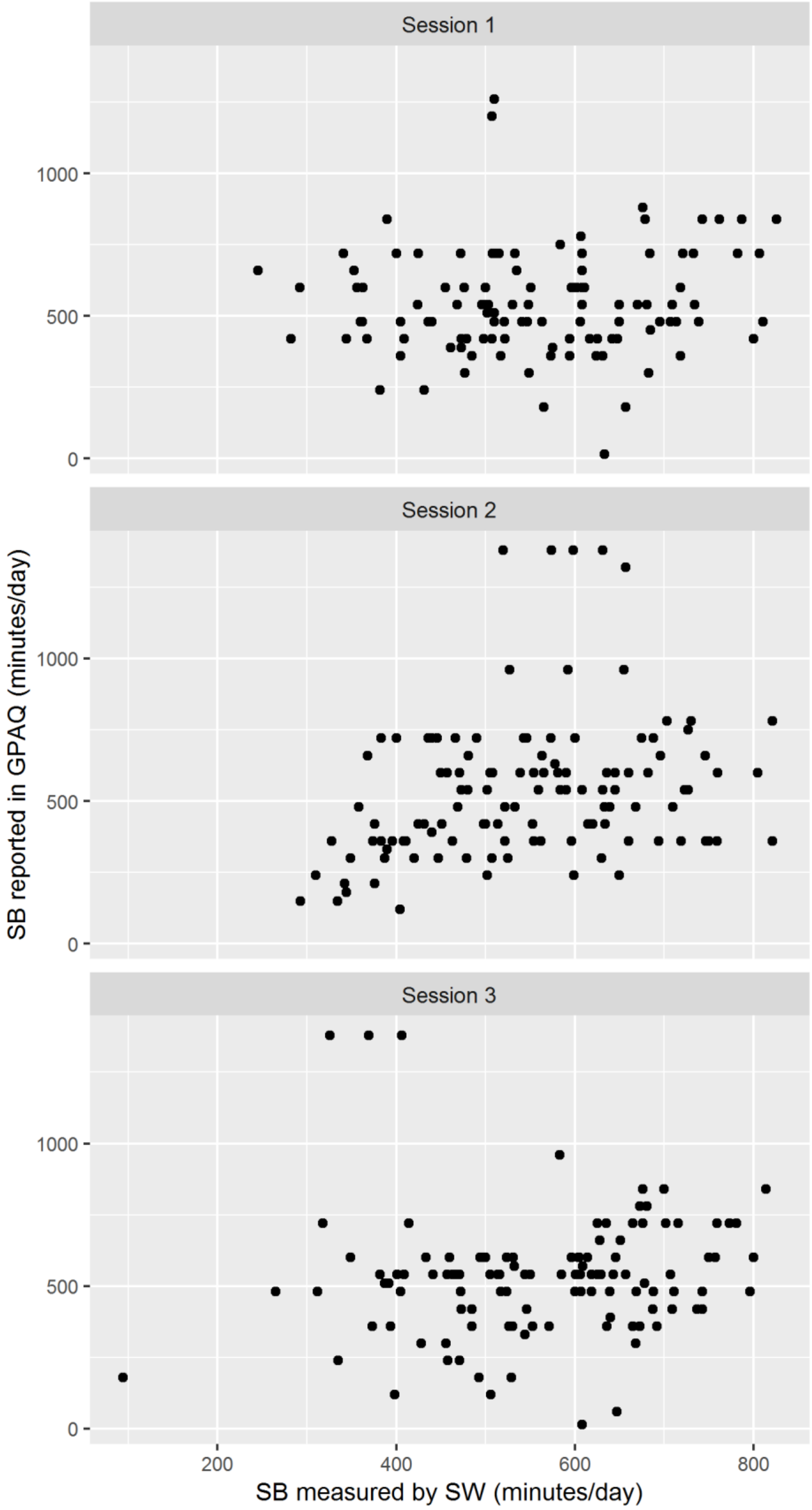

S2 Fig Sedentary minutes measured by the GPAQ in function of SB measured by the SenseWear. The Spearman correlation coefficients for session 1, session 2 and session 3 are respectively 0.09, 0.25 and 0.24 (overall rrm = 0.12). SW = SenseWear
