# Supplementary material for: Physical activity and sedentary behaviour in daily life: A comparative analysis of the Global Physical Activity Questionnaire (GPAQ) and the SenseWear armband
Source: PLoS One. 2017 May 16;12(5):e0177765. doi: 10.1371/journal.pone.0177765 (PMC5433749; doi:10.1371/journal.pone.0177765)
Supplement: S3 Fig — All percentage differences on the Y-axis are calculated by subtracting GPAQ from SenseWear results divided by their average. Moderate and vigorous intensity activities included influential observation. The red, dashed lines represent the mean difference and 95% limits of agreement excluding these observations. (PDF) [file pone.0177765.s006.pdf]

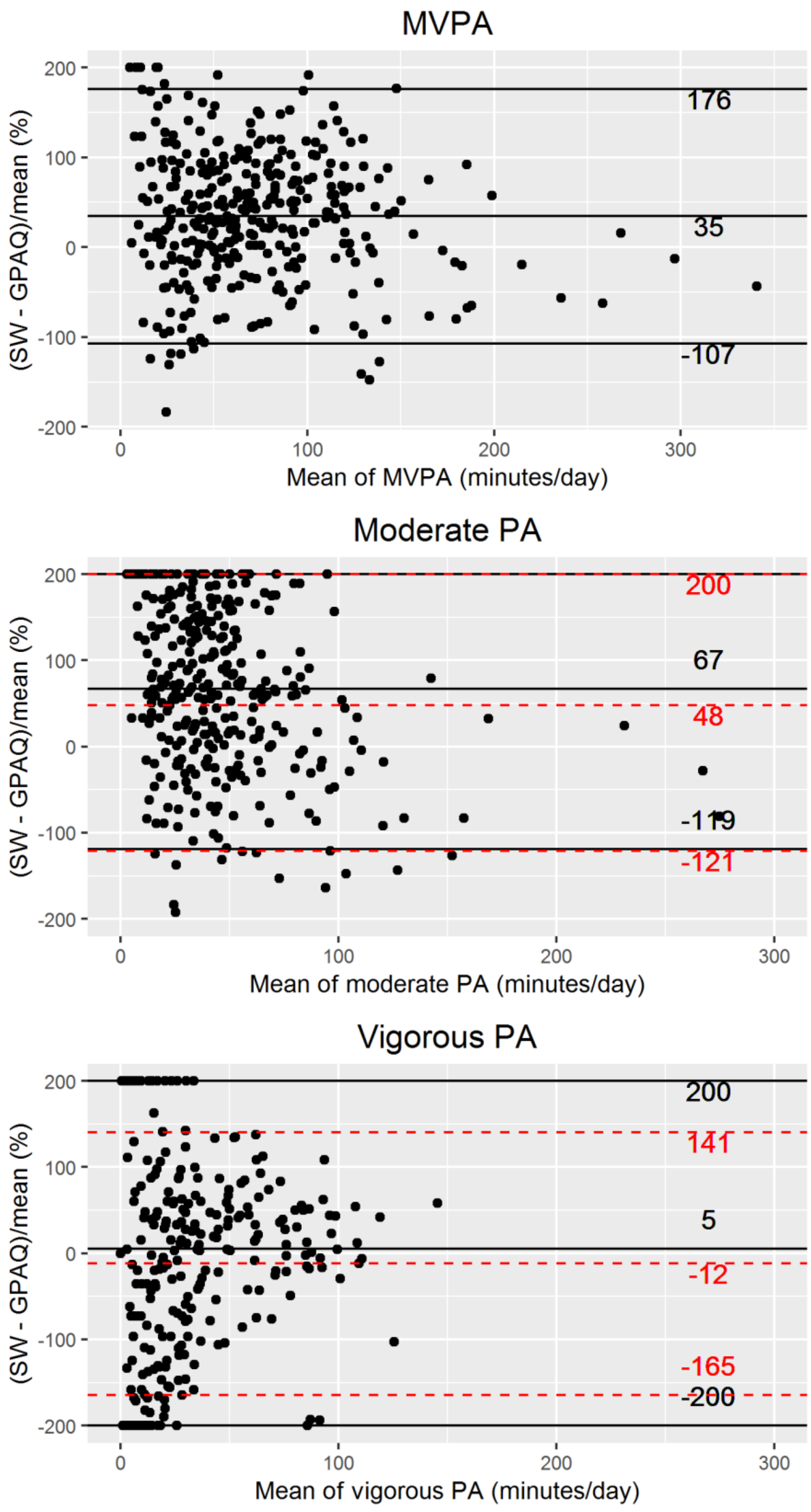

S3 Fig Bland-Altman plots comparing MVPA, moderate and vigorous time (minutes/week) measured by the SenseWear armband (SW) and the GPAQ. All percentage differences on the Y-axis are calculated by subtracting GPAQ from SenseWear results divided by their average. Moderate and vigorous intensity activities included influential observation. The red, dashed lines represent the mean difference and 95% limits of agreement excluding these observations.
